# A Case of Left Ventricular Thrombus and Herpetic Esophagitis in an Immunocompetent Patient With COVID-19

**DOI:** 10.7759/cureus.33640

**Published:** 2023-01-11

**Authors:** Ayman Mohamed, Harish Gidda, Shirin Zavoshi, Rabia Mahmood

**Affiliations:** 1 Internal Medicine, Ascension St. John Hospital, Detroit, USA

**Keywords:** coronavirus disease 2019, herpetic esophagitis, left ventricular thrombus, covid-19, immunocompetent adult, steroid treatment, hsv esophagitis, ventricular thrombus

## Abstract

Severe acute respiratory syndrome coronavirus 2 (SARS-CoV-2) infection has been associated with thrombosis, both venous and arterial, but the mechanism behind this coagulation is not fully understood. Several cases involving coronavirus disease 2019 (COVID-19)-positive patients with left ventricular thrombus (LVT), particularly in those with low ejection fraction, have been reported. This report describes a case of a 57-year-old male patient who presented to the hospital with altered mental status and a positive SARS-CoV-2 polymerase chain reaction (PCR) test. CT of the chest revealed the presence of an LVT, and transthoracic echocardiography showed a reduced ejection fraction and confirmed the presence of the thrombus. The patient also reported epigastric chest pain and several bloody bowel movements. A colonoscopy revealed internal hemorrhoids. An esophagogastroduodenoscopy revealed the presence of multiple esophageal ulcers, and biopsy results confirmed herpes simplex virus (HSV) infection. The patient had no history of organ or bone marrow transplant, long-term immunosuppressive therapy, or HIV infection. He was eventually discharged on apixaban for his LVT and acyclovir for his HSV esophagitis.

## Introduction

Severe acute respiratory syndrome coronavirus 2 (SARS-CoV-2) infection has been associated with both venous and arterial thrombosis, but the mechanism behind this coagulation is not fully understood. It is believed that damage to endothelial cells caused by SARS-CoV-2, as well as elevated levels of prothrombotic factors, increase the risk of thrombosis in these patients [1]. While left ventricular thrombus (LVT) is commonly found in patients after acute myocardial infarction [2,3], there have been a few reported cases in individuals who have tested positive for coronavirus disease 2019 (COVID-19), particularly in those with reduced left ventricular ejection fraction. In addition, the use of immunosuppressive medications during the course of COVID-19 infections may expose these patients to opportunistic infections that are usually seen in immunocompromised individuals [4-6]. SARS-CoV-2 itself may have an effect on the immune system increasing the risk of such infections [7]. In this report, we present a case of a 57-year-old COVID-19-positive patient with an LVT and a reduced left ventricular ejection fraction. Interestingly, the patient was also found to have herpes simplex virus (HSV) esophagitis, despite being immunocompetent.

## Case presentation

A 57-year-old man with schizophrenia presented to the hospital with altered mental status. An evaluation for stroke was initiated, but brain imaging did not reveal any large vessel occlusion or critical stenosis. A SARS-CoV-2 polymerase chain reaction test was positive.

At the time of presentation, the patient's blood pressure was 156/110 mmHg, respiratory rate was 34 breaths/minute, heart rate was 129 bpm, body temperature was 36.7°C, and oxygen saturation was 87% on room air. He received 2 L oxygen via nasal cannula, which improved his oxygenation levels. Upon examination, the patient had bilateral wheezing and pitting edema in the lower extremities.

**Figure 1:**
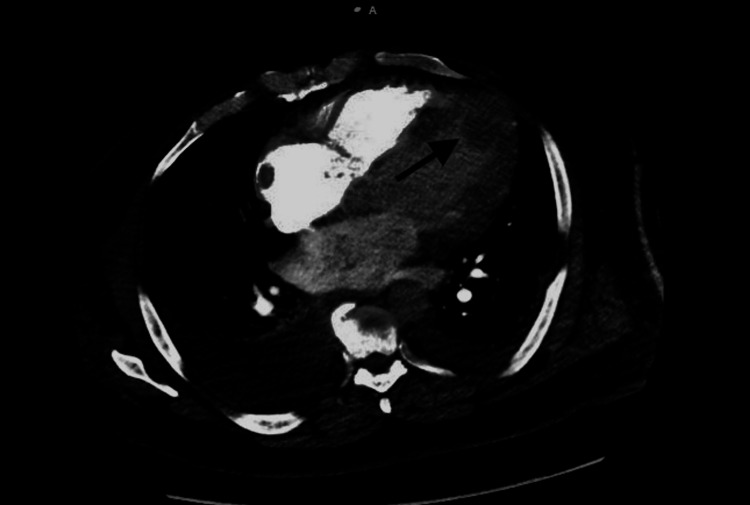
CT angiography of the chest showing a mass in the apical region

Upon laboratory testing, the patient's white blood cell count and procalcitonin level were found to be within normal limits. However, the patient had low bicarbonate levels and an increased anion gap. Mild elevations were observed in the patient's alanine aminotransferase (ALT) and aspartate aminotransferase (AST) levels. Lactate dehydrogenase (LDH), pro-brain natriuretic peptide (pro-BNP), lactate, and D-dimer levels were also elevated. A chest X-ray revealed decreased lung volumes with bilateral interstitial densities, and a CT angiography of the chest showed no evidence of pulmonary embolism. The CT angiography did, however, reveal patchy ground glass and interstitial opacities bilaterally, with scattered subsegmental consolidation, atelectasis, and large pleural effusions. Additionally, the CT angiography showed cardiomegaly and a mass in the apical region of the left ventricle (Figure 1).

**Figure 2:**
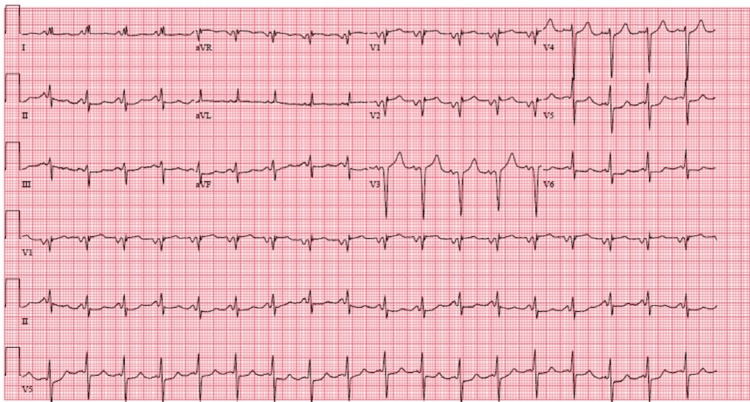
Electrocardiogram showing sinus tachycardia

A 12-lead electrocardiogram showed sinus tachycardia and no evidence of acute ischemic ST-T segment changes (Figure 2).

Imaging studies, including CT of the head with perfusion, head and neck CT angiography, and head CT without contrast, did not show any significant abnormalities. An echocardiogram performed as part of the stroke evaluation revealed a left ventricular ejection fraction of 20-30%, moderate global hypokinesis, and a mass attached to the apical segment of the anterior wall and apex, measuring 2.7 x 2.2 cm, which was believed to be an LVT given the patient's reduced ejection fraction and COVID-19-positive status. The patient had no previous echocardiograms for comparison. He was started on an intravenous unfractionated heparin drip and was given dexamethasone.

During the patient's first week of hospitalization, he experienced several bloody bowel movements and complained of burning epigastric chest pain. The heparin drip was discontinued and gastroenterology was consulted. The patient underwent a colonoscopy, which revealed colon polyps, mild diverticulosis, a rectal fissure, and internal hemorrhoids as the source of gastrointestinal bleeding. No active bleeding was observed during the procedures, and the heparin drip was restarted, and later switched to apixaban. An esophagogastroduodenoscopy (EGD) showed multiple esophageal and gastric ulcers with no active bleeding. Biopsies taken from the ulcer sites tested positive for herpes simplex. Further laboratory testing revealed positive HSV 1 and 2 IgM antibodies and HSV 1 glycoprotein G IgG antibodies, as well as negative HSV 2 glycoprotein G IgG antibodies. HIV-1 and 2 antibodies were non-reactive. The patient was treated with acyclovir 400 mg three times daily and intravenous pantoprazole 40 mg. After 21 days of hospitalization, he was discharged on oral anticoagulation therapy. A repeat echocardiogram was not performed, as the patient did not follow up.

## Discussion

The patient presented to the hospital with altered mental status, prompting an evaluation for a stroke. However, the stroke workup did not show any signs of acute ischemia. An echocardiogram was subsequently obtained to investigate possible intracardiac abnormalities, which revealed LVT and reduced ejection fraction with moderate global hypokinesis. The patient did not experience chest pain and his electrocardiogram did not reveal any acute ischemic changes, but he had several risk factors for thrombus formation, including COVID-19 infection and low ejection fraction. No previous echocardiograms had been performed and the patient had no known cardiac history.

LVT is a common occurrence in patients with non-ischemic dilated cardiomyopathy (DCM) and myocardial infarction [2,3]. In a study of 1302 patients with LVT, 39.2% were found to have non-ischemic DCM, while 29.8% had a history of myocardial infarction [3]. Thrombus formation is more likely to occur in the apical region due to the higher degree of blood stasis in this area [8]. Risk factors for left ventricular apical thrombus formation include male gender, prior myocardial infarction, apical aneurysm, and ischemic scarring [9].

SARS-CoV-2 binds to angiotensin-converting enzyme 2 (ACE2) receptors on endothelial and cardiac cells, leading to cell damage and the release of pro-inflammatory cytokines [10]. The COVID-19 infection also increases levels of prothrombotic factors such as fibrinogen, fibrin, factor VIII, von Willebrand factor activity, and D-dimer [1], leading to a hypercoagulable state and an increased risk of venous thromboembolism. The patient's low left ventricular ejection fraction, moderate global hypokinesis, and COVID-19 infection, all played a role in the development of the LVT.

The patient was also found to have HSV esophagitis, a rare condition in immunocompetent individuals that typically presents as an acute self-limiting illness [11,12]. It is more commonly seen in patients who have undergone bone marrow or solid organ transplants, are receiving aggressive immunosuppressants, or are living with HIV [13,14]. Both primary and reactivated HSV infections have been observed in individuals with COVID-19. A study of 83 COVID-19 patients admitted to the intensive care unit found that 21.7% had a reactivation of the mucocutaneous HSV [15]. Another cross-sectional study investigating the potential association between COVID-19 and HSV infection found that 35% of patients had an HSV infection, with 35.71% experiencing an initial HSV attack [7].

Our patient tested negative for HIV-1 and 2 antibodies but had positive HSV 1 and 2 IgM antibodies and HSV 1 glycoprotein G IgG antibodies, which were confirmed via biopsy. He had not undergone any transplant surgeries and was not taking long-term immunosuppressive medications, but had received a one-week course of dexamethasone at a dose of 6 mg daily. The use of dexamethasone during COVID-19 infections has been linked to the development of opportunistic infections such as HSV, aspergillosis, and mucormycosis [4-6]. It has also been suggested that the immune dysregulation and impaired cytotoxic T-cell function caused by SARS-CoV-2 may contribute to the increased frequency of HSV reactivation observed during COVID-19 infections [7].

## Conclusions

COVID-19 infections have been linked to several complications. Patients infected with the virus who have a low ejection fraction are at increased risk of developing intracardiac thrombus and may benefit from an echocardiogram to aid in the management of their condition. Additionally, these individuals may also be at risk of developing opportunistic infections that are typically seen in immunocompromised individuals. This may be due to the use of immunosuppressive medications during the course of the infection, or as a result of the virus's impact on the immune system. An appropriate workup for infectious causes can assist in the faster diagnosis and prompt treatment of these infections.
